# Cancer Incidence and Mortality Patterns in Hemodialysis Patients: A Descriptive Study

**DOI:** 10.7759/cureus.74145

**Published:** 2024-11-21

**Authors:** Amir Farah, Anna Tatakis, Wisam Abboud, Hala Saliba, Zaher Armaly

**Affiliations:** 1 Surgery, Medical College of Wisconsin, Milwaukee, USA; 2 General Surgery, Medical College of Wisconsin, Milwaukee, USA; 3 General Surgery, Nazareth Hospital Edinburgh Medical Missionary Society (EMMS), Nazareth, ISR; 4 Nephrology, Nazareth Hospital Edinburgh Medical Missionary Society (EMMS), Nazareth, ISR

**Keywords:** cancer, cancer mortality, cancer screening, carcinoma, dialysis, hemodialysis, nephrology, surveillance

## Abstract

Background

End-stage renal disease (ESRD) is a condition where the kidneys cease functioning, requiring renal replacement therapy such as dialysis. ESRD patients face numerous health challenges, including an elevated risk of developing malignancies. Factors contributing to this increased cancer risk include immune suppression, chronic inflammation, DNA repair deficiencies, and chronic viral infections.

Objective

This study aimed to describe the incidence and characteristics of malignancies, as well as associated risk factors, in patients undergoing hemodialysis.

Methods

This retrospective descriptive study included all patients receiving dialysis at the dialysis unit of our institution between 2012 and 2022 who were diagnosed with cancer. Cancer incidence and clinical characteristics were analyzed within this patient group.

Results

Out of 584 patients who underwent dialysis, 11 patients (2%) were diagnosed with cancer. The mean age of cancer patients was 76.5 years. Males accounted for 73% (n=8) of cancer cases. The most common malignancies identified were colorectal carcinoma (28%, n=3) and lung carcinoma (27%, n=3).

Conclusion

This study highlights the incidence of malignancies among ESRD patients on hemodialysis. Regular monitoring and early detection of malignancies in this high-risk population are crucial for improving outcomes.

## Introduction

End-stage renal disease (ESRD) is a severe and irreversible condition where the kidneys lose the ability to filter waste from the blood, necessitating renal replacement therapies such as dialysis. ESRD affects millions of people globally, with dialysis being the primary life-sustaining treatment for those who do not qualify for kidney transplantation. However, despite its life-prolonging benefits, dialysis introduces significant health risks. Patients undergoing hemodialysis face elevated risks of cardiovascular diseases, infections, bone disorders, and notably, cancer [1-4].

The link between ESRD and increased cancer risk has been well-documented [5,6]. Patients with ESRD are often immunocompromised due to long-term uremia, weakening the body’s ability to fight infections and malignancies [7]. In addition, chronic inflammation - commonly seen in dialysis patients - further exacerbates this risk [8]. Other contributing factors include oxidative stress, DNA repair deficiencies, and, in some cases, prolonged exposure to immunosuppressive drugs, especially among patients who have undergone kidney transplantation [9,10]. These biological factors collectively contribute to a predisposition to malignancies in ESRD patients.

Several studies have identified cancer types that occur more frequently in dialysis patients, including urological cancers such as renal cell carcinoma and bladder cancer, as well as gastrointestinal, lung, and skin cancers [11-14]. Some reports indicate that patients are most at risk of developing cancer within the first few years of dialysis, while others suggest a continuous increase in risk with prolonged dialysis exposure [15]. However, the specific risk factors and malignancies in dialysis patients may vary depending on geographic, demographic, and clinical factors [16].

Given the growing prevalence of ESRD and the extended life expectancy of dialysis patients due to advancements in care, studying cancer incidence and mortality in this population is of increasing importance. This descriptive study took place at the dialysis unit of our institution and investigated the incidence and mortality patterns of malignancies in patients undergoing hemodialysis. We aim to identify the types of cancers diagnosed, the time of diagnosis relative to dialysis initiation, and the clinical and demographic risk factors associated with cancer development.

## Materials and methods

This study employed a descriptive, retrospective design, conducted at the dialysis unit of the Nazareth Hospital Edinburgh Medical Missionary Society (EMMS), Nazareth, Israel. It included all patients undergoing hemodialysis between the years 2012 and 2022 who developed malignancies either during or after the initiation of dialysis. The aim was to investigate the incidence and types of malignancies within this patient cohort and explore associated demographic and clinical factors.

Study population and inclusion and exclusion criteria

The study population consisted of all hemodialysis patients treated at the dialysis unit during the specified period. Inclusion criteria were defined as patients who had undergone hemodialysis treatment for any length of time within the study period and had a confirmed diagnosis of cancer, either concurrent with or subsequent to their dialysis treatment. Exclusion criteria included patients under 18 years of age and pregnant women to ensure the generalizability and safety of the study findings within the adult population.

Data collection

Data were gathered from the hospital’s electronic medical record system and relevant databases, allowing for a comprehensive review of patient demographic and clinical data. Collected variables included demographic information (age, sex, and smoking status), dialysis-related parameters (duration of dialysis, dialysis frequency, and cause of kidney failure), clinical factors (comorbidities such as diabetes and hypertension), and laboratory results (serum creatinine, blood urea nitrogen (BUN), hemoglobin levels, etc.).

Cancer diagnoses were verified by two independent clinical extractors through a detailed review of patient medical records and relevant pathology reports, ensuring accuracy and consistency in diagnostic data. Any discrepancies in cancer diagnosis between the two extractors were resolved through discussion or consultation with a third senior clinician.

Statistical analysis

Statistical analyses were conducted using Statistical Package for the Social Sciences (IBM SPSS Statistics for Windows, IBM Corp., Version 24.0, Armonk, NY). Descriptive statistics were calculated to summarize baseline characteristics. Continuous variables such as age, duration of dialysis, and laboratory parameters were examined for normality and presented as means with standard deviations for normally distributed data, and medians with interquartile ranges for non-normally distributed data. Categorical variables, including gender, comorbidities, and cancer type, were summarized as frequencies and percentages.

Ethical considerations

This study was conducted in accordance with the ethical standards of the Institutional Review Board (IRB) of the Nazareth Hospital and with the 1964 Helsinki Declaration and its later amendments. Informed consent was waived due to the retrospective nature of the study, which involved only de-identified patient data and presented minimal risk to participants. Patient confidentiality was maintained throughout the study by using anonymized datasets and secure data storage protocols.

## Results

Incidence of cancer

Out of 584 patients undergoing dialysis at Nazareth Hospital between 2012 and 2022, 11 patients (2%) were diagnosed with cancer. The average age of cancer patients was 76.5 years. Gender distribution revealed that eight patients (73%) were male, while three patients (27%) were female.

Ethnicity and cancer

As predicted, the ethnic distribution in the cancer group showed that 10 patients (91%) were of Arab descent, which reflected the geographic position of the hospital, while one patient (9%) was of Eastern European origin.

Smoking and cancer

Among the cancer patients, three patients (27%) were smokers.

Other cancer risk factors

Hypertension was present in nine cancer patients (82%), and diabetes was more common in eight patients (73%). Ischemic heart disease was observed in seven cancer patients (64%), while hyperlipidemia was present in five patients (45%). Chronic obstructive pulmonary disease (COPD) was seen in three patients (27%), and two cancer patients (18%) were classified as obese.

Timing of cancer diagnosis

Among the cancer patients, three patients (27%) were diagnosed with cancer before starting dialysis, while eight patients (73%) were diagnosed after beginning dialysis. The average time between the initiation of dialysis and cancer diagnosis was 2.36 years.

Types of cancer

The most common cancers in this cohort were colorectal carcinoma (three patients, 28%) and lung carcinoma (three patients, 27%). Other types included glioblastoma, lymphoma, prostate cancer, cervical cancer, and esophageal carcinoma, each affecting one patient (9%).

Laboratory findings

Cancer patients had higher average levels of parathyroid hormone (PTH), with a mean of 399 pg/mL. BUN levels were elevated in cancer patients, with an average of 51 mg/dL. C-reactive protein (CRP) was also higher in cancer patients at 6.45 mg/L, indicating a heightened inflammatory state. Neutrophil levels were elevated at 73%, while lymphocyte levels were lower at 16% compared to typical values. Despite being ESRD patients, cancer patients exhibited lower creatinine levels, averaging 6 mg/dL (Table 1).

**Table 1:** Characteristics of cancer patients undergoing dialysis at the Nazareth Hospital between 2012 and 2022. The table includes the total number of patients, the incidence of cancer among them, and key demographic, clinical, and diagnostic data specific to cancer patients. The percentages reflect the proportion of cancer patients exhibiting each characteristic, with the two most common types of cancer highlighted (in parenthesis). COPD: chronic obstructive pulmonary disease

Characteristics	Values
Total patients undergoing dialysis	584
Patients diagnosed with cancer	2% (n=11)
Mean age of cancer patients	76.5 years
Gender distribution (Male)	73% (n=8)
Gender distribution (Female)	27% (n=3)
Most common cancer (Colorectal carcinoma)	28% (n=3)
Second most common cancer (Lung carcinoma)	27% (n=3)
Hypertension among cancer patients	82% (n=9)
Diabetes among cancer patients	73% (n=8)
Ischemic heart disease among cancer patients	64% (n=7)
COPD among cancer patients	27% (n=3)
Obesity among cancer patients	18% (n=2)
Patients diagnosed with cancer before dialysis	27% (n=3)
Patients diagnosed with cancer after dialysis	83% (n=8)

## Discussion

This study aimed to describe the incidence and characteristics of malignancies in patients undergoing hemodialysis for ESRD. Despite improvements in dialysis treatments, our findings underscore that cancer remains a significant health concern in this population, aligning with previous research on the increasing cancer burden in ESRD patients.

We observed a 2% cancer incidence in our cohort, which appears lower than the 4-10% cancer incidence typically reported in larger ESRD cohort studies. This discrepancy may be attributed to our relatively small sample size and limited follow-up period. Other studies with longer follow-ups have reported higher incidences, potentially capturing more late-onset malignancies that may not have been evident within the timeframe of our study. Future research should extend follow-up to better assess long-term cancer risk in dialysis populations.

Consistent with findings from other studies, colorectal and lung carcinomas were the most frequently diagnosed cancers in our cohort. This parallels cancer trends in the general population, where these malignancies are also prevalent. However, unlike some studies that report a higher incidence of urological cancers such as renal cell carcinoma and bladder cancer among dialysis patients, our cohort did not show a high prevalence of these types. This variation could be due to regional differences in cancer screening practices, genetics, or environmental factors. For instance, studies conducted in other geographic regions have reported elevated urological cancer risks, particularly in areas where specific genetic or environmental factors may contribute to these cancers. Additional research comparing cancer profiles across regions would help clarify these differences.

Regarding risk factors, hypertension, diabetes, ischemic heart disease, and COPD were common comorbidities among cancer patients in our study. This aligns with the findings of other studies that identify these conditions as prevalent in ESRD patients. Chronic conditions like hypertension and diabetes may contribute to cancer risk through mechanisms related to inflammation, immune suppression, and oxidative stress, all of which are heightened in ESRD. Our results are similar to the multi-comorbid profile usually described in ESRD populations, where the presence of multiple risk factors could compound cancer risk over time.

The laboratory findings in our study further support the hypothesis that chronic inflammation plays a role in cancer development among ESRD patients. Elevated levels of PTH, BUN, and CRP in our cancer patients suggest an ongoing inflammatory state and altered metabolic balance, conditions previously identified as contributors to increased cancer risk in ESRD populations. For instance, elevated CRP levels have been associated with cancer risk in both ESRD and non-ESRD populations, reinforcing the idea that chronic inflammation is a critical factor in malignancy development. Additionally, high PTH levels, which are linked to secondary hyperparathyroidism in ESRD patients, have been reported in other studies to correlate with cancer risk through mechanisms involving bone turnover and chronic inflammatory processes.

One notable finding in our study is that 83% of cancers were diagnosed after the initiation of dialysis, with an average latency of 2.36 years. This contrasts with reports from some studies suggesting that cancer risk is highest within the first six months of dialysis initiation. The prolonged latency observed in our study may indicate that long-term dialysis and its associated complications, such as chronic oxidative stress and immune suppression, contribute to cancer risk over time. This highlights the need for continued and comprehensive cancer surveillance in dialysis patients, even after the initial months of treatment.

While our study provides valuable insights, it is limited by its small sample size and descriptive design, which restricts the generalizability of the findings. Additionally, the retrospective nature of the study may have led to incomplete data, affecting the representativeness of the results. Larger, multi-center studies with longer follow-up periods are needed to validate our findings and explore the relationships between ESRD, dialysis, and cancer risk in more depth. Understanding these relationships will allow for the development of more targeted cancer screening and prevention strategies in ESRD populations.

## Conclusions

Although this is a descriptive study of a specific patient population, the study highlights the increased risk of malignancy in ESRD patients undergoing hemodialysis, particularly for colorectal and lung cancers. The findings emphasize the need for close cancer surveillance in this high-risk population. Larger, multi-center studies are needed to confirm these findings and to establish guidelines for cancer screening and management in dialysis patients.
